# In vitro progesterone production by luteinized human mural granulosa cells is modulated by activation of AMPK and cause of infertility

**DOI:** 10.1186/s12958-017-0295-9

**Published:** 2017-09-22

**Authors:** E. C. Bowdridge, M. W. Vernon, J. A. Flores, M. J. Clemmer

**Affiliations:** 1 Department of Biology West Virginia University, Morgantown, WV 26505 USA; 2 Department of Physiology and Pharmacology, West Virginia University, P.O. Box 4992, Morgantown, WV 26506 USA; 3 Department of Obstetrics and Gynecology, West Virginia University, Morgantown, WV 26506 USA

**Keywords:** Granulosa cells, Pregnancy, Luteal insufficiency

## Abstract

**Background:**

Mural granulosa cells from IVF patients were provided by the West Virginia University Center for Reproductive Medicine in Morgantown, WV. The effect of adenosine monophosphate activated protein kinase (AMPK) activation, primary cause of infertility, age, BMI, and pregnancy outcome on production of progesterone were examined separately.

**Methods:**

Isolated mural sheets from IVF patients (*n* = 26) were centrifuged, supernatant discarded, and the pellet re-suspended in 500 μl of DMEM/F12. Mural granulosa cells were plated at 10,000 cells/well in triplicate per treatment group with 300 μl DMEM/F12 media at 37 °C and 5% CO2 in a humidified incubator to permit luteinization. Four days after initial plating, cells were treated with either an AMPK inhibitor, DM; an AMPK activator, AICAR; or hCG. Cells were cultured for 24 h after treatment when medium was collected and frozen at −20 °C until assayed for P4 by radioimmunoassay.

**Results:**

The AMPK activator, AICAR, inhibited P4 production (*P* < 0.001), whereas the AMPK inhibitor, DM, did not affect basal P4 (*P* < 0.05). Progesterone production increased when cells from patients whose primary cause of infertility was a partner having male infertility were treated with hCG compared to control (*P* = 0.0045), but not in patients with other primary infertility factors (*P* > 0.05). Additionally, hCG increased P4 production in patients between the ages 30–35 (*P* = 0.008) and 36–39 (*P* = 0.04), but not in patients ages 25–29 (*P* = 0.73). Patients with normal BMI had increased P4 production when treated with hCG (*P* < 0.0001), however there was no change in P4 production from cells of patients who were overweight or obese (*P* > 0.05). Cells from patients who became pregnant to IVF had greater P4 production when stimulated with hCG than those who did not become pregnant when compared to controls (*P* > 0.05).

**Conclusions:**

Understanding how AMPK activation is regulated in ovarian cells could lead to alternative or novel infertility treatments. Human mural granulosa cells can serve as a valuable model for understanding how AMPK affects P4 production in steroidogenic cells. Additionally, when stimulated with hCG, P4 production by mural granulosa cells differed among infertility type, age, BMI, and pregnancy outcome.

**Electronic supplementary material:**

The online version of this article (10.1186/s12958-017-0295-9) contains supplementary material, which is available to authorized users.

## Background

Two distinct populations of granulosa cells exist within the ovulatory follicle, the mural granulosa cells that line the follicle and the cumulus granulosa cells that surround the oocyte. After ovulation, mural granulosa cells remain in the follicle while cumulus granulosa cells are expelled from the follicle with the oocyte [19]. The mural granulosa cells that remain in the ovulatory follicle lose their ability to proliferate and to produce estradiol. Under the influence of the LH surge which induced the ovulation, the mural cells undergo an epithelial-mesenchymal transition that leads to the formation of the large luteal cells (LLC) of the corpus luteum [CL], which secrete high amounts of progesterone [P4] [6]. In the presence of human chorionic gonadotropin [hCG] from a conceptus, the CL is rescued from luteolysis and maintains its production of P4, which is essential for the maintenance of pregnancy. Insufficient luteal rescue may lead to decreased P4 production that can lead to pregnancy loss. Luteal insufficiency has been estimated to be as high as 28% in couples with recurrent pregnancy loss [14, 15]. A better understanding of insufficient luteal rescue could improve the clinical diagnosis and treatment of early pregnancy loss.

In multiple species, sufficient energy is required for P4 production [23, 24]. Adenosine monophosphate activated protein kinase [AMPK] is a key regulatory protein for energy balance, and activation of AMPK results in a shift from anabolic to catabolic processes [11]. In rat granulosa cells, P4 was directly inhibited by AMPK activation [25]. Various subunits of AMPK have also been identified in the granulosa cells of small and large bovine follicles [23]. 5-Aminoimidazole-4-carboxamide-1-β-4-ribofuranoside [AICAR] is a well-established, cell-permeable agent, which both directly activates AMPK alloterically and promotes phosphorylation of AMPK [7]. Treatment with AICAR (7.5 mM) significantly decreased basal P4 production in bovine CL in vitro [4]. In contrast, dorsomorphin dihydrochloride (DM) is a potent, selective, cell-permeable and reversible AMPK inhibitor [17]. Treatment with DM inhibited AMPK activation induced by AICAR [22] or metformin [12].

The objectives of this project were to investigate the role of AMPK, a known intracellular mediator of energy balance, and a patient’s primary cause of infertility on the production of P4 in luteinized human granulosa cells.

## Methods

### Patients

Mural and cumulus granulosa cells from patients (*n* = 26) undergoing egg retrieval after ovarian stimulation for in vitro fertilization [IVF] were provided by the WVU Center for Reproductive Medicine in Morgantown from April to December 2012. The age, BMI and primary cause of infertility were random as all patients with granulosa cells collected at oocyte retrieval were included in the study. Primary cause of infertility was assigned based on severity of the problem contributing most to preventing a successful pregnancy for each patient, with preference given to any issues with the female. Any endocrine related factor, such as endometriosis, was prioritized as the primary cause of infertility over any anatomical factor, such as tubal blockage, because these types of endocrine conditions were more likely to affect the granulosa cells collected for experiments. Patients were designated as male factor patients if there were no indications of female infertility and the male partner presented with an abnormal semen evaluation.

Daily or twice-daily injections of recombinant follicle stimulating hormone [FSH] began on d2 or d3 of the cycle and continued until ovulation was induced with hCG. One of the following preparations of FSH was used: Bravelle (Ferring Pharmaceuticals, Parsippany, NJ), Gonal F (EMD Serono, Rockland, MD), or Follistim (Merck, Whitehouse Station, NJ) in addition to Menopur (Ferring Pharmaceuticals Inc. Parsippany, NJ), which was used for every patient. Patients returned at least every other day for sonographic and hormonal monitoring of follicular growth beginning four days after initiation of stimulation. To prevent premature ovulation, gonadotropin releasing hormones [GnRH] antagonist injections (Ganirelix, Merck, Kenilworth, NJ) began when follicles reached at least 14 mm in diameter. Ovulation was induced with hCG injection (10,000 IU; Novarel, Ferring Pharmaceuticals, Parsippany, NJ) when at least two follicles were ≥18 mm in diameter, followed by oocyte collection 34 h later.

### Isolation and culture of primary human GCs

Sheets of mural granulosa cells were distinguished by anatomical structure and manually separated from cumulus granulosa cells by experienced embryologists following oocyte retrieval. Isolated mural sheets from each patient in G-MOPS Plus media (Vitrolife, Göteborg, Sweden) were centrifuged at 10,000 rpm for 5 min at room temperature. Supernatant was discarded, and the pellet was re-suspended in 500 μl of DMEM/F12 (Invitrogen, Grand Island, NY) with 20% (*v*/v) fetal bovine serum, insulin/transferrin/selenium B (1000; 555; 0.67 mg/L respectively; Gibco, Grand Island, NY), gentamicin/amphotericin (10 μg/ml-0.25 μg/ml; Gibco, Grand Island, NY), and penicillin/streptomyocin (100 I.U./ml, 100 μg/ml; Life Technologies, Grand Island, NY). Viable cell number was determined using trypan blue dye exclusion (Gibco, Grand Island, NY) and a hemocytometer. All patients had cell viability >90% and cells were plated at 10,000 cells per well in 96-well flat bottom culture plates with 300 μl DMEM/F12 media (NUNC, Scientific Laboratory Supplies, Wilford, Nottingham, UK) at 37 °C and 5% CO_2_ in a humidified incubator to permit luteinization and cell attachment. Media was changed every 24 h whereby 150 μl of spent media was replaced with fresh DMEM/F12 plus serum. Peak progesterone levels in human mural granulosa cells have been shown to occur on day 4 of culture [1], as was the case during preliminary testing of our culture conditions of days of luteinization ranging from 1 to 7 (data not shown), and therefore used as the experimental day. Four days after collection, cells were transferred to serum free DMEM/F12 media, and treatments were administered. Every treatment was tested in triplicate for each patient and media pooled for P4 by radioimmunoassay [RIA] as previously described [20]. Control values for each patient were obtained by culturing cells in triplicate with no hormones. This basal progesterone production, which was obtained on the same day as treatments, was used as a baseline for each patient, and treatments with pharmacological agents or hormones were compared to baseline within patient.

### Experiment 1

Treatments were based on previous studies in bovine luteal steroidogenic cells [3] and mural granulosa cells. The medium for the control group contained 0.1% dimethylsufoxide (DMSO, Pierce Rockport, IL), the solvent used for DM and AICAR. Mural granulosa cells isolated from patients presenting with tubal or male factor infertility were used in experiment 1 to control for any confounding female endocrine effect. Mural granulosa cells were treated with DM (109, 10.9, 1.09, 0.109 μm/L, Tocris, Bristol, UK) or AICAR (7.5, 0.75, 0.075, 0.0075 mM, Tocris, Bristol, UK) to evaluate the roles of inhibiting or stimulating AMPK, respectively, in luteinized mural granulosa cell production of P4. Cells were cultured for 24 h and medium was removed and frozen at −20 °C until assayed for P4.

### Experiment 2

Cells were treated with PGF_2α_ (0.1 μg/ml; Cayman Chemical, Ann Arbor, MI), or human hCG (5 IU/ml; Novarel, Ferring Pharmaceuticals, Parsippany, NJ), or the combination of the two (PGF_2α_ + hCG). Treatment concentrations of PGF_2α_, hCG, or PGF_2α_ + hCG and incubation times were based on previous studies with bovine luteal steroidogenic cells [3] and preliminary studies testing 24, 48, and 96 h incubation times in mural granulosa cells, respectively. Cells were cultured further for 24 h after treatment at which time medium was removed and frozen at −20 °C until assayed for P4. Main cause of infertility (male factor, endometriosis, tubal factor, and unexplained), age (<30, 30–35, 36–39 years of age), BMI (normal = 18.5–24.9, overweight = 25–29.9, obese ≥30), and pregnancy outcome (non-pregnant and pregnant) were analyzed.

### Statistical analysis

Concentrations of P4 were tested for normal distribution via the Shapiro-Wilk test. All data were transformed using natural logarithm. All other experiments were analyzed using one-way ANOVA and Dunnett’s post hoc test. Data were analyzed using JMP and SAS software (JMP®, Version Pro 11, SAS Institute Inc., Cary, NC, Copyright ©2013; SAS®, Version 9.3, SAS Institute Inc., Cary, NC, Copyright ©2002–2010). Differences were considered significant when *P* < 0.05. Data throughout are depicted as the mean ± SE.

## Results

### General patient information

Patients presented randomly with the following as their main cause of infertility: male factor (*n* = 5), endometriosis (*n* = 6), tubal factor (*n* = 12; *n* = 11 naturally occurring tubal issues and n = 1 tubal ligation), and other/unexplained (*n* = 3). They ranged in age from 25 to 39 years of age (mean age 33.0 ± 0.70) and were classified as <30 (*n* = 4), 31–35 (*n* = 15), or ≥36 (*n* = 7). Patients had a BMI range of 18.9 to 44.3 (mean BMI 25.0 ± 1.1) and were classified as normal 18.5–24.9 (*n* = 17), overweight 25–29.9 (*n* = 6) or obese ≥30.0 (*n* = 3). Most patients (*n* = 13) were pregnant after the IVF cycle was completed; 10 did not become pregnant and the remaining patients (*n* = 3; 1 no fertilization and 2 planned embryo cryopreservation) did not receive a fresh embryo transfer at the end of the IVF cycle. Information about the general response of these patients to stimulation and IVF are recorded in Table 1.

**Table 1:** General patient information

Patient Information	n	Range	Mean
Age	26	25–39	33 ± 3.58
BMI	26	19–36	24.96 ± 5.50
Gravidity	26	0–9	1.42 ± 2.44
Total Gonadotropins (IU)	26	450–4200	1613 ± 899
Stimulation Days	26	7–12	9.19 ± 1.20
Day 3 FSH (mIU/mL)	20	4–13	7.32 ± 2.16
E2 at trigger (pg/mL)	26	364–3669	1678 ± 837
P4 at trigger (ng/mL)	26	0.43–2.63	1.13 ± 0.68
Total oocytes retrieved	26	2–23	8.4 ± 4.7
MII oocytes retrieved	26	2–18	6.5 ± 3.8
Fertilization rate (%)	26	0–100	82.6 ± 24.5
Division rate (%)	26	67–100	95.8 ± 8.8

Ranges and means for gravidity, stimulation, hormonal profiles, oocytes retrieved during the IVF cycle, fertilization rate of MII oocytes, and division rate of fertilized oocytes

### Experiment 1

**Fig. 1:**
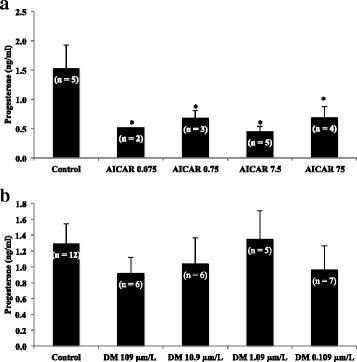
Effect of altering AMPK on progesterone production by granulosa cells. **a** The AMPK activator, 5-Aminoimidazole-4-carboxamide-1-β-4-ribofuranoside [AICAR], inhibited progesterone production by human luteinized mural granulosal cells at all dosages tested. In **b**, the AMPK inhibitor, dorsomorphin [DM], did not affect basal progesterone production. Asterisks indicate a difference of (*P* < 0.05) from control value. All treatments were plated in triplicate for each experiment. N values for each treatment group are given in parenthesis above each column

To more deeply understand production of progesterone in luteinized granulosa cells, the pathway involved in energy regulation was manipulated. The AMPK activator, AICAR, inhibited P4 production by human luteinized mural granulosa cells at all dosages tested (*P* < 0.001; Fig. 1a). In Fig. 1b, the AMPK inhibitor, DM, did not affect basal progesterone production (*P* < 0.05).

### Experiment 2

Within cause of infertilty, no significant differences in P4 were seen when granulosa cells were treated with PGF_2α_ or PGF_2α_ + hCG in any infertility types (data not shown), with the exception of male infertility, (control P4 = 0.77 ± 0.24 ng/ml vs. GF_2α_ = 1.56 ± 0.68 ng/ml; *P* < 0.05) Additionally, no significant differences in P4 were seen when granulosa cells were treated with PGF_2α_ or PGF_2α_ + hCG in any age or BMI category, or pregnancy outcome (data not shown).

**Fig. 2:**
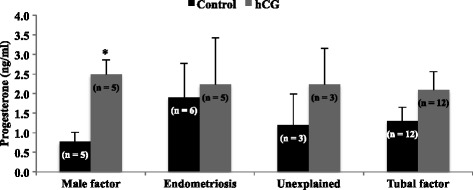
Progesterone production by granulosa cells from patients with different causes of infertility. Progesterone production by granulosa cells from patients with male factor, endometriosis, other/unexplained, and tubal infertility before and after treatment with PGF_2α_ (0.1 μg/ml) or hCG (5 IU/ml). Asterisks indicate a difference of (*P* < 0.05) from control value within infertility group. N values for each treatment group are given in parenthesis above each column

There was an increase in P4 production over baseline in cells treated with hCG and collected from patients that presented with no known female/male infertility factor (0.77 ± 0.24 ng/ml vs. 2.49 ± 0.38 ng/ml; *P* = 0.0045; Fig. 2), but not in patients who presented with endometriosis (1.91 ± 0.87 ng/ml vs. 2.23 ± 1.2 ng/ml), other/unexplained (1.19 ± 0.80 ng/ml vs. 2.23 ± 0.93 ng/ml) or tubal factor (1.30 ± 0.36 ng/ml vs. 2.10 ± 0.46 ng/ml; *P* > 0.05; Fig. 2).

**Fig. 3:**
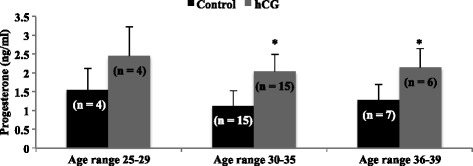
Progesterone production by granulosa cells from patients of different ages. Patients who ranged in age from 25 to 29 years old did not response to hCG with a significant increase in progesterone production. However, other age groups (30–35 and ≥36 years old) did have a significant increase in progesterone when stimulated with hCG. Asterisks indicate a difference of (*P* < 0.05) from control value within age ranges. Cells were treated with PGF_2α_ (0.1 μg/ml) or hCG (5 IU/ml). N values for each treatment group are given in parenthesis above each column

Additionally, patient age influenced the ability of hCG to stimulate a significant increase in P4. Production of P4 increased following treatment with hCG for patients in the age range of 30–35 (1.23 ± 0.40 ng/ml vs. 2.03 ± 0.46 ng/ml; *P* = 0.008) and 36–39 (*n* = 7; 1.41 ± 0.45 ng/ml vs. 2.37 ± 0.53 ng/ml; *P* = 0.04), but not in patients in the ranges of 25–29 (*n* = 4; 1.52 ± 0.75 ng/ml vs. 2.65 ± 0.91 ng/ml; *P* = 0.73; Fig. 3).

**Fig. 4:**
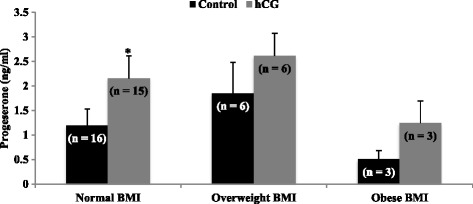
Progesterone production by granulosa cells from patients with different body mass indexes. Patients with normal body mass index [BMI] (18.5–24.9) had a significant increase in progesterone production when treated with hCG (*P* < 0.0001). Cells of patients who were categorized as overweight (BMI 25–29.9) or obese (BMI >30) did not have an increase in progesterone production when stimulated with hCG (*P* > 0.05). Asterisks indicate a difference of (*P* < 0.05) from control value with BMI categories. Cells were treated with PGF_2α_ (0.1 μg/ml) or hCG (5 IU/ml). N values for each treatment group are given in parenthesis above each column

Patients with normal BMI had a significant increase in P4 production when treated with hCG (1.29 ± 0.34 ng/ml vs. 2.25 ± 0.46 ng/ml; *P* < 0.0001; Fig. 4), but granulosa cells of patients who were categorized as overweight or obese did not have a significant increase in P4 production when stimulated with hCG (1.85 ± 0.63 ng/ml vs. 2.61 ± 0.46 ng/ml and 0.50 ± 0.18 ng/ml vs. 1.24 ± 0.45 ng/ml, respectively; *P* > 0.05; Fig. 4).

**Fig. 5:**
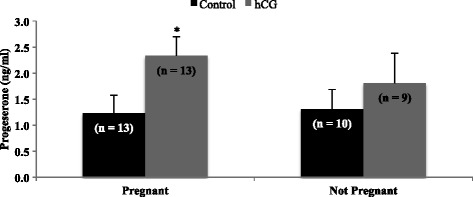
Progesterone production by granulosa cells in non-pregnant versus pregnant patients. Progesterone production in mural granulosal cells was affected by pregnancy status post in-vitro fertilization (IVF). Luteinized mural granulosa cells from patients who became pregnant to the IVF cycle produced significantly more progesterone when stimulated with hCG than those who did not become pregnant (2.4 vs. 1.8 ng/ml; *P* < 0.05). Asterisks indicate a difference of (*P* < 0.05) from control value with pregnancy outcome. Cells were treated with PGF_2α_ (0.1 μg/ml) or hCG (5 IU/ml). N values for each treatment group are given in parenthesis above each column

Figure 5 demonstrates that granulosa cells from patients who became pregnant to the IVF cycle had significantly increased P4 production compared to controls when stimulated with hCG (2.4 ± 0.37 ng/ml; *P* = 0.0018 vs. control; Fig. 4) whereas those who did not become pregnant did not (1.8 ± 0.58 ng/ml; *P* > 0.05 vs. control; Fig. 5).

## Discussion

Activation of AMPK, a key regulator of energy homeostasis, decreased basal P4 production in bovine [23] and rodent [25] granulosa cells, whereas AMPK increased P4 in galline [24] granulosa cells. In agreement with the findings in cattle and rats, treatment with AICAR resulted in a significant decrease in basal P4 at all concentrations tested in experiment 1. Similarly, AICAR treatment resulted in a decrease in P4 production in the mature bovine CL [4]. This decrease in P4 could be explained, at least in part, by a decrease in key enzymes such as 3βHSD, P450scc, or StAR, all of which have been shown to be decreased after treatment with AICAR in rat granulosa cells [25]. These experiments show that the AMPK pathway is inhibitory to basal P4 production in luteinized human granulosa cells in the early stage of luteinization and presumably even more so with further luteal maturation.

In contrast to the effect of AMPK activation, no difference in P4 production were seen when mural cells were treated with an AMPK inhibitor, DM. The concentrations of DM chosen for Experiment 1 were based on studies in bovine CL, in which DM significantly decreased P4 production following stimulation with PGF_2α_ (Bowdridge, unpublished results). However, a greater concentration of DM may be needed to elicit a change in basal P4 production in human mural granulosa cells. Additionally, because DM is an AMPK inhibitor cells may need to first be stimulated with hCG, PGF_2α_, or another AMPK activator prior to DM treatment in order to see a reduction in P4 production. Due to the limited number of granulosa cells that are collected from each patient, these experiments were unable to be performed simultaneously. It is also possible that the effect of inhibition of AMPK in granulosa cells differs between species such as cattle and humans, much the same way as AMPK activation increases P4 in the chicken, but not in rats or chickens. Therefore, it appears that stimulated, but not basal P4 secretion, is regulated by AMPK in human mural granulosa cells.

Data from Experiment 2 support the conclusion that infertility type affects the ability of luteinized human mural granulosa cells to respond in vitro to the luteotropic factor, hCG. If these cells likewise failed to respond to hCG in vivo it could lead to luteal insufficiency or even early regression of the CL, resulting in pregnancy loss. Our hypothesis is that the in vivo environments in which granulosa cells develop contribute to their ability to respond to hCG as they transition into a luteal phenotype. Progesterone production after stimulation with hCG in granulosa cells from endometriosis, unexplained, and tubal factor patients was not different than basal P4 production. Therefore, these patients were unable to respond to stimulation by hCG, despite having normal basal P4 levels. However, as expected, granulosa cells from patients with partners having male infertility exhibited a significant increase in P4 production when compared to control values. Clinically, all patients undergoing IVF treatments are regularly given empirical supportive P4 after embryo transfer, which eliminates the need for the patient’s granulosa cells to increase P4 in response to embryo derived hCG to sustain a pregnancy. However, using mural granulosa cells to identify each patient’s ability to naturally respond to hCG could allow for administration of lower levels of exogenous P4. There is recent evidence that supplemental P4 after the first positive β-hCG pregnancy test following IVF may not be necessary to achieve a live birth [16]. Elimination of unnecessary P4 supplementation could reduce cost and time requirements for patients undergoing IVF.

A significant increase in P4 was seen in mural cells of those women who became pregnant following IVF. In contrast, P4 production in the hCG-stimulated treatment group was not different from controls in the mural cells of women who did not become pregnant following IVF. Failure of these cells to secrete high levels of P4 in vivo is similar to a condition known as luteal phase defect [LPD]. In LPD there is only a brief elevation in P4 secretion (typically <11 days) after ovulation, and LPD is reported to occur in up to 20% of women [2]. The mean peak P4 level for women with short luteal phases was 1.8 ng/ml compared to the mean peak P4 level of 7.6 ng/ml for women with normal luteal phase length [21]. Interestingly, LPD has been associated with endometriosis [8], polycystic ovarian syndrome [PCOS], obesity [10], and aging [18]. These conditions differ significantly in their etiology, however all have the potential to influence the hormonal environment in which granulosa cells develop and their ability to function as luteal cells. A short luteal phase may be due to a defect in the process of luteinization or P4 production, or both. Experiments 1 and 2 were performed prior to any luteal phase supplementation in these patients, and therefore the P4 production would be a representation of their innate ability to respond to physiological doses of hCG. The inability of these cells to respond could potentially be explained by impaired cholesterol transport mechanisms within the ovary, or a deficiency in LH receptors. In addition, gene expression for STAR, CYP11A1, and 3βHSD were all significantly reduced in ewes that exhibited a short luteal phase during the non-breeding season [5]. Stimulation of mural granulosa cells with hCG and measurement of P4 levels on day 4 of culture, prior to day 5–6 blastocyst transfers, would determine if hCG response was predictive of pregnancy success independent of embryo quality.

It was not surprising that P4 production by mural granulosa cells was affected by age and BMI, both of which have previously been shown to alter steroid production in vivo [9, 26]. Surprisingly, the age group that did not have an increase in P4 production was the youngest age group. It should be noted, however, that this group had the largest numerical increase in P4 when stimulated with hCG. However, the fact that granulosa cells from two of the four patients responded which lead to highly variability, several different causes of infertility (two tubal factor, endometriosis, male) and the small population size (*n* = 4) could separately or collectively account for the lack of difference within this age group. In contrast, it was encouraging to see that mural cells from older patients responded to luteotropic hCG, suggesting that these cells are capable of increases in P4, which are needed for a successful pregnancy.

Patients with BMIs in either the overweight or obese range did not respond to hCG with increased P4 production. However, due to the small patient number in the overweight (*n* = 6) and obese groups (*n* = 3) these results should be interpreted cautiously. Nonetheless, combining the data from these two groups to increase patient number still did not result in a significant increase in hCG stimulated P4 production (*p* = 0.11). The interrelationship between nutritional status and reproduction is complicated, and the mechanisms connecting the two are not well defined. It will be critical to ascertain if the mural cells of overweight and obese patients have insufficient substrate to produce P4 when stimulated or if they are resistant to hCG despite adequate substrate. In a recent study using non-human primates, weight gain directly impaired P4-secretion, which was accompanied by decreased mRNA expression for LH receptor, P450scc, STAR and 3βHSD within the CL, suggesting that obesity can directly affect CL function [13].

## Conclusions

In summary, understanding how drugs such as AICAR, DM, and other AMPK activators alter steroidogenic pathways in ovarian cells could lead to alternative or novel infertility treatments. Human mural granulosa cells serve as a valuable model for understanding how AMPK and its downstream targets affect P4 production in steroidogenic cells. Additionally, when stimulated with hCG, P4 production by mural granulosa cells differed among main infertility type, age, BMI, and pregnancy outcome. Investigating differences in basal and stimulated P4 concentrations amongst such patients may also give insight into luteal steroidogenesis in an infertile population. In addition to basal and stimulated P4 production, mRNA and protein levels of key enzymes such as P450scc, StAR, and 3βHSD should be evaluated across age, BMI, pregnancy outcome, and infertility type. Critically evaluating similarities and differences in granulosa cell P4 production between the previously mentioned factors has the potential to increase successful full-term gestations in patients undergoing IVF, and decrease the incidence of miscarriages induced by luteal insufficiency.

## Additional file

Additional file 1: Progestereone data from experiments 1 and 2. (XLSX 16 kb)

## Abbreviations

3βHSD: 3-Beta-Hydroxysteroid Dehydrogenase
AICAR: 5-Aminoimidazole-4-carboxamide-1-β-4-ribofuranoside
AMPK: Adenonsine monophosphate activated protein kinase
BMI: Body mass index
CL: Corpus luteum
DM: Dorsomorphin
DOR: Diminished ovarian reserve
FSH: Follicle stimulating hormone
GnRH: Gonadotropin releasing hormone
hCG: Human chorionic gonadotropin
IRB: Institutional Review Board
IVF: In-vitro fertilization
LH: Luteinizing hormone
LPD: Luteal phase defect
P4: Progesterone
P450scc: Cholesterol side chain cleavage enzyme
PCOS: Polycystic ovarian syndrome
RIA: Radioimmunoassay
StAR: Steroidogenic acute regulatory protein
